# Case report: Thoracic schwannoma as a diagnostic pitfall in both ^18^F-Choline PET/CT and ^18^F-FDG PET/CT

**DOI:** 10.3389/fonc.2024.1467344

**Published:** 2024-10-08

**Authors:** Karim Amrane, Coline Le Meur, Pierre Alemany, Clémence Niel, David Renault, Inès Peillon, Valentin Tissot, Ronan Abgral

**Affiliations:** ^1^ Department of Oncology, Regional Hospital of Morlaix, Morlaix, France; ^2^ Inserm, UMR1227, Lymphocytes B et Autoimmunité, Univ Brest, Inserm, LabEx IGO, Brest, France; ^3^ Department of Radiotherapy, University Hospital of Brest, Brest, France; ^4^ Department of Pathology, Ouest-Pathologie, Brest, France; ^5^ Department of Radiology, University Hospital of Brest, Brest, France; ^6^ Department of Nuclear Medicine, University Hospital of Brest, Brest, France; ^7^ UMR Inserm 1304 groupe d'étude de la thrombose de bretagne occidentale (GETBO), Instituts Fédératifs de Recherche (IFR) 148, University of Western Brittany, Brest, France

**Keywords:** prostate cancer, thoracic schwannoma, 18 F-choline PET/CT, MRI, FDG-PET/CT

## Abstract

We report increased ^18^F-FDG uptake in the right posterior mediastinal region in a 70-year-old woman following the discovery of a mass in the aftermath of a bronchitis episode. We also report increased ^18^F-Choline uptake in the right posterior mediastinal region in a 66-year-old man with newly discovered prostate cancer, which may indicate the presence of mediastinal metastases. Both patients had a thoracic MRI showing an intense gadolinium enhancement in the same region, consistent with thoracic schwannomas, which were subsequently proven histologically. This case highlights that schwannoma is a diagnostic pitfall in both ^18^F-FDG and ^18^F-Choline PET/CT.

## Introduction

A schwannoma is a generally benign soft tissue tumor that arises from Schwann cell myelin in the peripheral nervous system. It can be difficult to distinguish from malignant soft tissue tumours. The number of malignant peripheral nerve sheath tumors is relatively small, and the incidence rate is 3-10% of soft tissue sarcomas (1). Thoracic schwannomas are usually located in the posterior mediastinum (2, 3) and are often large in size (4), but rarely intrapulmonary (5, 6). On imaging, schwannomas appear in the path of a nerve, are well limited, often oval and single. MRI shows low to intermediate signal intensity on T1-weighted images, high signal intensity on T2-weighted images and intense enhancement of solid components on gadolinium-enhanced imaging. Regions of very high T2-weighted signal intensity correspond to cystic degeneration and may show only peripheral enhancement or no enhancement associated with very high signal intensity. Overall, the larger the schwannoma, the more heterogeneous it appears on all sequences (including gadolinium-enhanced images) due to cystic degeneration, hemorrhage or both (3). The metabolic characteristics of schwannomas on ^18^F-FDG PET-CT are not well described in the literature to differentiate benign or malignant forms. There is no association between SUV and benign or malignant schwannomas (7, 8) moreover lesions with heterogeneous FDG activity had higher SUVmax and more frequent internal non-enhancement on MRI (9). Radiolabeled choline is becoming a promising tracer in the diagnosis of glial tumors due to its low distribution rate in normal white and gray matter, leading to a high background signal-to-noise ratio compared with ^18^F-FDG PET/CT (10). Thus, patients with suspected recurrent glial tumors have been shown to have a more clearly defined abnormal accumulation on ^18^F-Choline PET/CT (11).

## Case description

**Figure 1:**
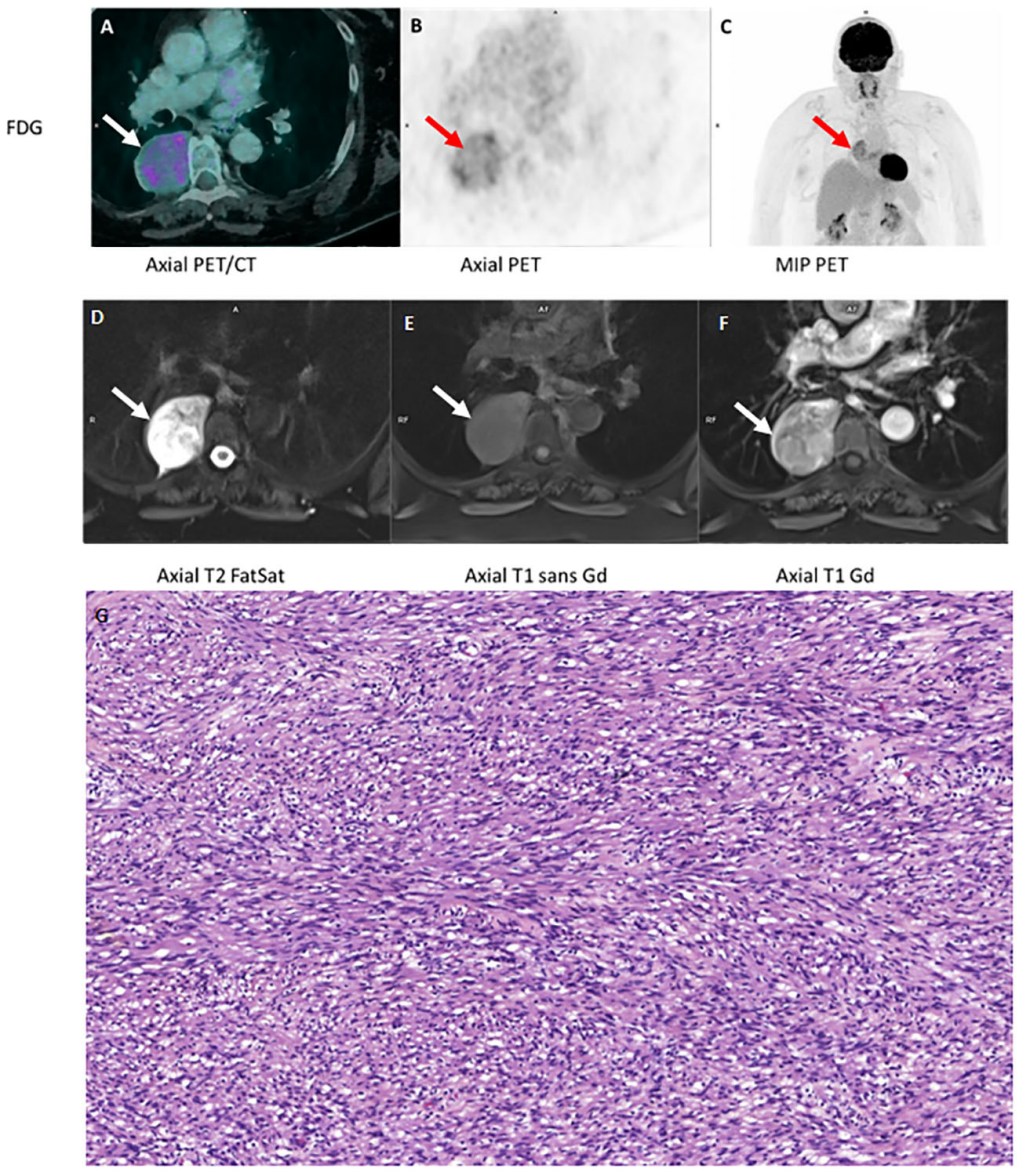
FDG PET/CT **(A, B, C)** MRI **(D, E, F)** and **(G)** (schwanomma histology).

We first present a 70-year-old woman with no significant past medical history other than 15 pack-years of smoking. Following severe bronchitis, a right paravertebral mass measuring 62 x 49 mm was seen on chest radiography. This corresponded to a hypermetabolic lesion (SUVmax 6.7) on ^18^F-FDG PET, without locoregional extension. **Figures 1A–C**.

**Figure 2:**
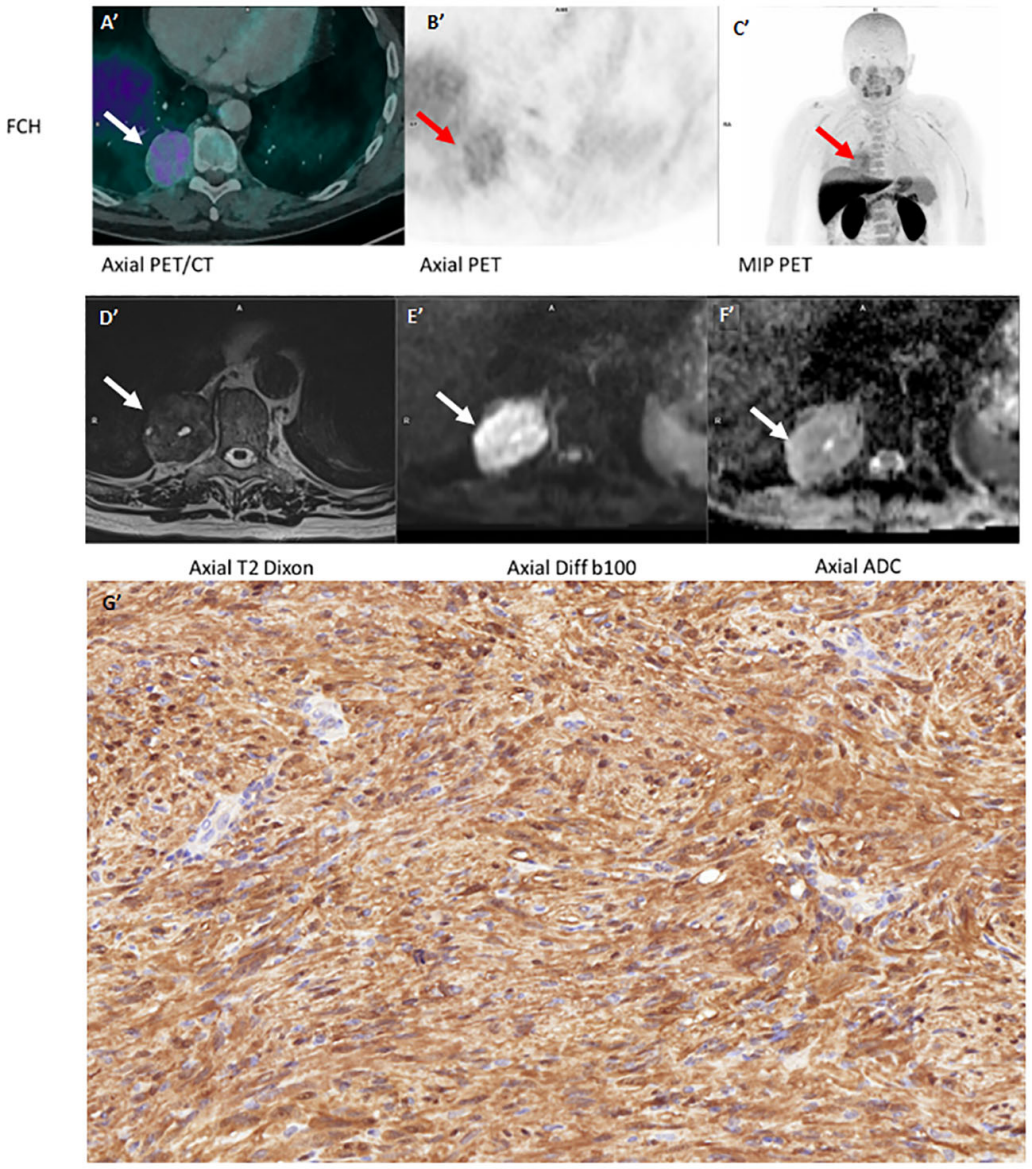
FCH PET/CT **(A', B', C')** MRI **(D', E', F')** and **(G')** (schwanomma histology).

We then present the case of a 66-year-old man with a particular history of Gleason 8 prostatic adenocarcinoma. ^18^F-Choline PET/CT for disease extension revealed a right posterobasal lung mass almost 5 cm long with moderate uptake (SUVmax 6.4) at the posterior pleural contact. **Figures 2A’-C’**.

Complementary MRI was performed for both two lesions (panel of different MRI sequence both two cases). It showed same characteristics: heterogeneous T2 hypersignal with some microcystic areas, T1 isosignal to muscle and moderate diffusion restriction; gadolinium injection resulted in early, intense and relatively homogeneous enhancement outside cystic (or myxoid) areas. **Figures 1D–F** and **Figures 2D’-F’**.

Both patients (**Figure 1G**’ and **Figure 2G**’) were operated on with complete excision in favor of a benign schwannomatous nerve lesion, with a tumor proliferation rate (Ki-67 index) of 3% for both lesions. (**Figure 1G**: In the pleura, tumor characterised by spindle cells intermingled in bundles. Absence of cytonuclear atypia. Magnification x20. **Figure 2G’**: High magnification image (x40) of the tumor expressing the positive S100 protein in immunochemistry with strongly and diffusely signal over the entire lesion).

## Discussion

^18^F-FDG PET/CT is used to differentiate between benign and malignant tumors (12). However, false-positive and false-negative results with ^18^F-FDG PET/CT are relatively common. ^18^F-FDG uptake in benign schwannomas varies considerably, and benign schwannomas with high FDG uptake can easily be misdiagnosed as malignant. Indeed, even with low levels of Ki-67, the lesion may be avid for ^18^F-FDG, as there is no correlation between Ki-67 expression and ^18^F-FDG uptake in schwannomas (13), and this avidity for ^18^F-FDG could be due to overexpression of GLUT1 or GLUT3, although this is not certain as studies give different results (14, 15).

However, ^18^F-Choline avidity is less controversial in suspected schwannomas as it is a precursor of phosphatidylcholine, one of the main components of the cell membrane. Its specific pathophysiological pathway allows it to be used as a marker of cell proliferation in PET, helping to detect tumors that synthesize a lot of cell wall, such as prostate cancer (16). Schwannomas are composed of myelin, the precursor of which is phosphatidylcholine (17), which explains their detection on ^18^F-Choline PET/CT. Although it remains difficult to distinguish between a metastasis and a schwannoma in the case of ^18^F-Choline uptake, the unusual location of this uptake in the context of a prostate cancer metastasis could more easily point to another etiology, such as a benign tumor.

## Conclusion

It seems difficult to conclude a diagnosis of schwannoma on the basis of PET-CT SUV characteristics alone, as their SUVmax seems to vary enormously. There are also several other causes of false positives, such as infectious and inflammatory processes.

Although promising, ^18^F-Choline PET/CT cannot play a role in the management of newly diagnosed glial lesions, as it is a non-tumor-specific tracer. Biopsy remains the gold standard for accurate diagnosis.

## Data Availability

The original contributions presented in the study are included in the article/supplementary material. Further inquiries can be directed to the corresponding author.
